# Report of a Few Surgical Cases, with Remarks on Practical Points

**Published:** 1895-06

**Authors:** J. B. S. Holmes

**Affiliations:** Atlanta, Ga.


For Texas Medical Journal.

### REPORT OF A FEW SURGICAL CASES, WITH REMARKS ON PRACTICAL POINTS.

BY J. B. S. HOLMES, M. D., ATLANTA, GA.

[Read at Dallas Meeting Texas State Medical Association.]

I HEREWITH beg to submit a condensed report of a few surgical cases, all done in private practice:

CASE No. 1.—*Nephrectomy for Sarcoma.*—On April 6th, 1894, Mary N., aged 13 months, was brought to me for examination. Her family history good, free from tuberculosis, cancer, or syphilis. She was rather small for her age, but in good health up to early in January, three months before I saw her. At that time she became irritable, and suffered considerable digestive disturbance, accompanied with loose bowels. Her mother, at this time, detected a small tumor, about the size of a hen's egg, in the right lumbar region. She consulted her family physician, who diagnosed the case as enlargement of the liver. The child continued under his treatment until the latter part of March. She was then carried to my friends, Drs. R. L. Harbin, of Calhoun, and W. C. Nixon, of Nannie, Georgia. Both gentlemen kindly referred the case to me. After a thorough examination, my diagnosis was given as sarcoma of the right kidney. The child was brought to my sanatorium on the 9th instant, three days later, for operation. A dose of oil was given, with instructions that nothing be allowed for nourishment except its mother's milk, and nothing be taken on the following morning. The next day, after an all-over bichloride bath, the abdomen was thoroughly cleansed with oil of turpentine, tincture of green soap, and then again using bichloride.

Nephrectomy was done by making an incision, beginning near the lower border of the 8th rib, and extending down on the outer side of the linea semilunaris to near Poupart's ligament. As the tumor was quite large, pushing the peritoneum far over to the left, I had hoped to do an extra peritoneal operation, but in making my incision and reaching the capsule, I found it so firmly adherent to the peritoneum as to be unable to separate it without doing great and serious damage, and perhaps lacerating this membrane. Again, on account of the immense size of the growth, I was unable to enucleate it without going into the abdominal cavity. I was forced to make a transverse incision, extending from about the middle of the longitudinal one, three inches in each direction.

After considerable difficulty, the capsule was detached from the peritoneum, and the tumor turned out. It was my purpose to have ligated the artery and vein together, and the ureter separately, but as considerable time had been consumed in breaking up the adhesions, I ligated the ureter and vessels en masse, using silk. The peritoneum was closed, without flushing, by a running suture of fine silk. A drainage tube of rubber was placed in the lower angle of the wound, which was thoroughly cleansed with sterilized water, before closing. The incision was closed with deep sutures of silkworm gut, which, however, did not include the peritoneum. It was dressed with sterilized gauze, with bichloride cotton on it. The tube was removed at the end of forty-eight hours. The patient was put to bed in good condition, having lost not more than an ounce of blood. At the the end of twelve hours, her temperature had reached 100. The following day, at 3 p. m., twenty-eight hours after the operation, her temperature had reached 102½, considerable distention of the abdomen, but no gastric disturbance had shown itself.

I gave her one grain each of calomel and soda, with instructions that one-half grain of each be given every four hours until three grains had been given, or until the bowels had freely moved. The first dose acted freely within three hours, she passing six immense lubricoid worms within the next twelve hours. The following day her temperature was normal, and so continued until the fourth day, when there was a slight elevation, reaching 100. I examined the dressings, and found some redness at one stitch, which had been drawn too tight. This was removed, and a little pus escaped, when her temperature, in six hours, dropped to normal, and so continued. On the sixth day, every alternate stitch was removed; the remainder the day after. With the exception of the one stitch-hole abscess, the incision healed without suppuration. No opium was given, and she took no nourishment except her mother's milk. After the first twenty-four hours, she had water ad libitum. The tumor, with the kidney, weighed 3¼ pounds. The kidney rested almost as a flattened shell upon the surface of the tumor. The patient returned to her home in three weeks. She continued in excellent health until March of the present year (1895), when she died of acute pneumonia.

Case No. 2.—Capt. D., aged 56, Alabama, came to my sanatorium October 20th, intensely weak, pulse 130, temperature 102, swelling in right iliac region as large as a fœtal head. He stated that two weeks before he was seized with a severe pain about three inches to the right and a little below the umbilicus, accompanied with high fever, and vomiting. He was treated for liver disease, until he became so alarmed about his condition that, as ill as he was, he left home and came to Atlanta, consulting my friend, Dr. E. H. Richardson, who promptly referred him to me. Upon examination, I diagnosed perforative appendicitis, with suppuration. His condition was so extreme, that I operated within an hour after he came into the sanatorium. He was given a hypodermic of strychnia 1-30 of a grain; the skin over the abdomen was cleansed thoroughly with oil of turpentine, green soap, and then a bichloride solution, 1 to 100. An incision was made from McBurney's point, extending two inches downward. Just as my knife entered the abscess sack, he coughed, and the pus spurted at least three feet high. About a quart of extremely fetid pus was discharged. The cavity was thoroughly irrigated with boiled water, after which peroxide of hydrogen was freely used, and the cavity packed with iodoform gauze. I did not search for the appendix, which I am sure had been entirely destroyed by the suppurative process. The dressings were removed each day, and peroxide of hydrogen used, and the packing of iodoform gauze reintroduced. His improvement was continuous, and he left the sanatorium November 19th, with the wound entirely healed, and he in excellent condition. His health has continued good, as letter dated April 11th states.

This case illustrates how wisely and well nature takes care of her subjects. This old gentleman had traveled more than three hundred miles, with only a thin membrane between him and eternity; for had this ruptured, and the contents of the pus sack gone into the peritoneal cavity, how soon would septic peritonitis have ended his life.

Case No. 3.—Mrs. C., age 21, Nullip, Kentucky. Married two years. Began menstruation at fifteen, and without pain until marriage. Up to marriage, had been in excellent health,—weighed 143 pounds. Soon after marriage, suffered an attack of pelvic peritonitis, and from that time until she came under my observation, her health had steadily given way, and when I saw her she only weighed 90 pounds. Suffered with insomnia, no appetite, poor digestion, constipation, frequent and painful micturition, backache, intense pain in the pelvis, most severe on the left side; constant bearing down sensation while standing or walking. On examination, found the uterus firmly fixed, and left ovary and tube very much enlarged, also fixed, extremely sensitive and tender. The right also fixed and enlarged, but not to the same extent. An ovariotomy was done October 20th, and upon opening the abdomen I found the ovaries and tubes adherent to everything within their reach. After much difficulty, the adhesions were broken up, and the ovaries and tubes removed. The abdomen was thoroughly irrigated with boiled water at a temperature of 105, and glass drainage used, the tube remaining forty-two hours. Her recovery was uninterrupted, and she is to-day in perfect health; free from pain, good appetite, perfect digestion, sleeps well, and weighs 125 pounds. The left ovary and tube were both filled with pus, the right ovary cystic, and the right ovary filled with blood. The cause of the trouble in this case was, in my opinion, beyond a doubt, gonorrhœa, the deadliest of all enemies to the health of women. I report the case as a typical one, to show the terrible ravages that it can commit in so short a time. I could report a number of such cases.

Case No. 4.—Mrs. H., aged 32, multipara. North Carolina. Menstruation profuse, and intensely painful; severe headache, insomnia, acute pain in back, bearing down sensation in pelvis, inability to stand or walk. Had not walked a step in nine months. A confirmed opium habitue. Upon examination, I found the right ovary prolapsed, and fixed in Douglas' cul-de-sac, and I think the most sensitive of any that I have ever touched. The left ovary was prolapsed and cystic, about the size of a hen's egg; uterus retroflexed, and considerably enlarged. On November 22d, I removed both ovaries and tubes, elevating the uterus by shortening the round ligaments, which were included in the ligatures encircling the ovarian pedicle. She left the sanatorium December 24th, free from pain, and walking without discomfort. She is now (April 1st), as a recent letter states, attending to her household duties, and in perfect health. Was entirely free from the opium habit before leaving the sanatorium, and has so continued. This patient had had local treatment for nearly two years before coming under my care.

Case No. 5.—Mrs. D., aged 36, multipara. Florida. Menstruation regular, but with great pain, violent headache, insomnia, no appetite, poor digestion, constipated, nausea, intense pain in back and lower abdomen, extending down the thighs. unable to walk, and had been confined to her bed for six months; an invalid for fifteen years. Upon examination, I found floating kidney on the right side, uterus enlarged and retroflexed; right ovary the size of a guinea's egg, firmly fixed in Douglas' sac; the left ovary prolapsed, cystic, sensitive.

Ovariotomy was performed December 18th, removing both ovaries and tubes, and elevating uterus. Her convalescence from the operation was satisfactory in every particular. On February 5th, the right kidney was anchored. I made an incision about two and one-half inches from the spine, beginning at the lower border of the twelfth rib, and extending three and one-half inches in an oblique direction towards the crest of the ilium. When the kidney was exposed, the capsule was incised, and folded back one-fourth inch on each side. Four silkworm gut sutures were passed through the muscles, the fatty capsules of the kidney, and through the kidney tissue. Twelve strands of silkworm gut were placed on the kidney, and brought out at the upper and lower angles of the wound. The kidney was pressed firmly in position by the hand of my assistant, then three additional sutures of silkworm gut were passed from the skin through the muscles, fatty capsules, and kidney tissue. The four deep sutures were drawn moderately tight, and tied, cut close, and the cutaneous wound closed by the three deep sutures and several superficial ones. A dressing of sterilized gauze and absorbent cotton was applied over the wound, and a pad of gauze was applied over the kidney, on its anterior aspect, and held in position by rubber straps passing half round the body. On the eighth day, the silkworm gut threads were removed, and also the sutures. Perfect union had taken place throughout the entire length of the wound. The abdominal pad was worn for about thirty days. At this date (April 9th), the kidney remains firmly fixed. She has gained wonderfully in strength and flesh, is relieved of all pain, eats and sleeps well, walks wherever she pleases about the sanatorium, and will return to her home Saturday, the 20th instant. This patient had been a victim for ten years of iodine, iodized phenol, carbolic acid, nitrate of silver, "*et id omne genus.*"

Ether was administered to all the foregoing cases, except

[NOTE.—Dr. Holmes reported five other cases, but owing to length of paper, we have omitted them, and give in full his comments.—ED.]

numbers 1 and 4, to whom chloroform was given. I believe if the secondary effect of ether upon the kidneys was studied, we would find as many or more deaths attributable to it than to chloroform, both primarily and secondarily. The risk of ether is undoubtedly greater secondarily than primarily. Many deaths are caused by it that are attributed to other causes. When carefully used and properly administered, I believe chloroform to be quite as safe, and it is certainly much more satisfactory. The struggling of the patient, when being anæsthetized, is done away with, and the horrible sick stomach, which usually follows the the use of ether, rarely occurs with chloroform. The return to consciousness, after taking chloroform, is rarely accompanied with vomiting or severe nervous manifestations. I am daily more inclined to the use of chloroform, and expect, in the near future, to see the tide of professional opinion turn in its favor. Before administering it, if my patient is at all weak, I always give a hypodermic of 1-30 to 1-20 of a grain of strychnia. There is still great diversity of opinion among many able surgeons as to the relative safety of the two anæsthetics in the presence of kidney disease. Some even claim that chloroform produces more renal trouble than ether. I shall always, until experience proves to the contrary, give chloroform the preference in any diseased condition of the kidneys, unless there are potent reasons why ether should be used. I never operate in any case where albumen is shown in the urine in any quantity, without a thorough test for urea and a microscopical examination for casts, unless it be one of great emergency. If the quantity of urine is near normal, and the quantity of urea not notably deficient, hurried operations may be done, even if we have albumen, and some casts. If, however, the quantity of urine passed is small, and the amount of urea notably deficient, I would only operate for pus accumulations or very large tumors producing severe pressure symptoms. Where diagnosis can be satisfactorily made of chronic intersticial nephritis, which is a very difficult thing to do, all operative procedures are contra-indicated. Where I have them under control, I like for my patients to flush the kidneys for three or four days preceding operation with Lithia water, and I always use it for two or three weeks afterwards. It is always well, before surgical procedure, in those cases where you have the preparation of the patient, to see that the skin is clean and in an active condition.

We are all in the habit, I am sure, of giving too much ether and chloroform. If properly used, after the patient is thoroughly anæsthetized, very little is required to keep up complete anæsthesia.

I find strychnia in surgical work the most reliable of all heart tonics. Where the patient comes under my care early enough, unless she is very strong, I always give 1-30 of a grain of strychnia every four to six hours for three or four days before the operation, and if she shows evidences of shock afterwards I give it freely, watching for its physiological effects, and continuing it for several days until the pulse is sufficiently strong. I consider it far better as a stimulant and heart tonic than brandy, which, however, I also use when required. For surgical shock I have very little faith in digitalis, it has always been disappointing to me. Case No. 8 has impressed me with the importance of always going prepared to use saline infusions, which have, I think, a most valuable place in the treatment of shock from loss of blood.

My experience with catgut, both for ligatures and sutures, has been unsatisfactory. I now never use it, except where I do a trachelorrhaphy and perineorrhaphy at the same sitting. It is hard to sterilize, is soft, slips easy, and therefore hard to tie with any assurance of safety; and besides it is frequently absorbed earlier than we would like.

I have used many of the prepared foods, different broths, lime water and milk, peptonized milk, etc., after abdominal operations; but, if I was restricted to one article of diet for these cases I would select buttermilk. I find many patients, who in health, could not use it at all, after a laparotomy take it with the greatest relish, and it is extremely rare that I find one with whom it disagrees.

As a hypnotic I have found nothing so satisfactory as ten to fifteen grains of trional, repeated in an hour if sleep is not produced. The use of it has not been followed by any unpleasant secondary symptoms.

As a purgative, after an abdominal operation, I am more in the habit of using epsom salts than any other remedy. Experience proves it be the most satisfactory to me of all laxatives. I usually give it in three drachm doses, repeating every three hours until the bowels move. I have used it hypodermically in two cases only: one satisfactorily; in the other no effect was noticed.

I have always deprecated the use of opium, especially in abdominal work, and have used it with great care and caution. An increasing experience convinces me that those cases where it is not given at all do much better, and their convalescence is more satisfactory in every respect. While the pain after abdominal sections is frequently severe, it rarely continues but a short while. Where the pain is so intense and threatens to exhaust the patient from continued suffering, I think it advisable to use it in moderation. I think many of the cases of bowel obstruction, whether due to paresis, lymph bands or adhesions, are traceable to the use of opium. I am convinced that the adhesions in case No. 8, where the abdomen was reopened, were due to the paralyzed or quiescent condition of the bowels, so rendered by the two doses of morphia as given after the operation. While all peristalsis was arrested and the bowel lying quietly against the ovarian pedicle, the lymph was rapidly pouring out and the constriction produced. Had the peristalsis kept up, no adhesions would have occurred. Just here let me enter a plea for the early reopening of the abdomen in all cases of diagnosed or strongly suspected post-operative obstruction. Do not let your patient die without giving her a chance for life; reopen the abdomen, find the obstruction and relieve it.

In my laparotomy cases, where it has been necessary to elevate the uterus for retroflexion or retroversion, I have done so by shortening the round ligaments, by including them in the ligatures that encircle the ovarian pedicle. In the few cases in which I have done this, the uterus has remained well forward, while it is still allowed free movement. It is not fixed against the bladder, causing great irritability of that organ, that ventrofixation sometimes does.

In doing ovariotomy, I, in no case, operate simply for nervous and reflex symptoms. I operate only for a diseased condition that I am sure can be relieved only by surgical procedure. Careful examination, post-operative, have confirmed my opinion in every instance, in all the cases upon which I have operated.

While I believe in conservative measures, the most radical are often the most conservative. I am ready to admit that the abdominal cavity has often been ruthlessly invaded and organs removed for nervous symptoms, due entirely to other causes, and for which they were in no wise responsible. On the other hand, tinkering gynecology, I am sure, is responsible for many diseased tubes and ovaries that can only be cured by removal. The introduction of various caustic remedies into the uterus, causing destruction and sloughing of the mucous membrane, with a cervical canal in many cases previously constricted, or so rendered by the applications, that the decomposing masses can not pass out. What follows? A septic endometritis, which soon extends to the tubes and ovaries, producing trouble that nothing but the knife will relieve. This must be resorted to, or the poor woman is frequently doomed to permanent invalidism; a condition sometimes worse than death. Many of the cases that the surgeon is censured for using the knife upon are caused by such treatment.

I do not condemn all local treatment. Properly directed, I believe there are many conditions, where it is not only admissible, but demanded. Often in these conditions cures are made, especially where the local treatment is supplemented by constitutional measures, for oftentimes local troubles are clearly the results of constitutional vices. I do condemn, however, in the strongest terms, the reckless, haphazard and indiscriminate introduction of the various caustics into the uterus: certainly none should be introduced without first providing for thorough drainage. The want of cleanliness on the part of many physicians, when making vaginal and uterine examinations, is often a potent factor in causing utering and pelvic disease. Not only should the hands and instruments be clean, but the vagina and cervix should be thoroughly cleansed and disinfected before an instrument of any kind is passed into the uterus. How many of you have seen your professional brother swab the uterus with all sorts of medicaments, using dirty cotton on an equally unclean applicator, never cleansing the vagina and cervix, and even passing a sound into the uterus that had been passed in perhaps a dozen others without coming in contact with soap and water. While the uterine sound is a valuable instrument in its place, there are but few instances where it is required. I believe it has done more damage to women than all other instruments combined, and it not only has been, but is still, too indiscriminately used. Stem pessaries have made many victims for the surgeon's knife. With the possible exception of the uterine sound, they have done more harm than any other instrument or appliance.

Understand me, I do not think that every diseased ovary should be removed; far from it. I decline operation in many cases where the patient is anxious for it. I try to be conscientious and operate only where the disease is so far advanced that it has or is likely to render the woman an invalid, and is incurable by any other means. There are many other points I would like to discuss, but the length of this paper forbids.

